# Thrombotic Events and Stroke in the Year After COVID-19 or Other Acute Respiratory Infection

**DOI:** 10.3201/eid3114.250630

**Published:** 2025-12

**Authors:** Caroline Q. Pratt, Alexandra F. Dalton, Emily H. Koumans, Abraham Agedew, Fatima Coronado, Elizabeth A. Lundeen, Rebecca C. Woodruff, Jason P. Block, Mark Weiner, Lindsay Cowell, Jonathan D. Arnold, Sharon Saydah, PCORnet Network Partners

**Affiliations:** Centers for Disease Control and Prevention, Atlanta, Georgia, USA (C.Q. Pratt, A.F. Dalton, E.H. Koumans, A. Agedew, F. Coronado, E.A. Lundeen, R.C. Woodruff, S. Saydah); Harvard Pilgrim Health Care Institute & Harvard Medical School, Boston, Massachusetts, USA (J.P. Block); Weill Cornell Medicine, New York, New York, USA (M. Weiner); The University of Texas Southwestern Medical Center, Dallas, Texas, USA (L. Cowell); University of Pittsburgh School of Medicine, Pittsburgh, Pennsylvania, USA (J.D. Arnold)

**Keywords:** Long COVID, COVID-19, SARS-CoV-2, stroke, thrombosis, viruses, respiratory infections

## Abstract

Previous studies have documented an increased risk for thrombotic events 30 days after COVID-19 infection, but less is known about this risk beyond 30 days or compared with risk after other infectious acute respiratory illnesses (ARIs). By using PCORnet data from April 1, 2022–April 30, 2023, we compared the incidences of thrombotic events in the year after COVID-19 illness with other ARI diagnoses in hospitalized and nonhospitalized patients. Overall, the risk for any thrombotic event was higher among patients with COVID-19 compared with patients with other ARIs (incidence ratio 1.63; p<0.05). Nonhospitalized patients with COVID-19 had a 73% increased risk for a thrombotic event in the year after acute illness compared with nonhospitalized patients with ARI (p<0.05). The increased risk for thrombotic events in the year after COVID-19 emphasizes the need for stroke awareness for patients and healthcare professionals.

Stroke and thrombotic events are known sequelae of respiratory viral illnesses, including influenza and COVID-19 (*1*–*5*). Since the onset of the COVID-19 pandemic, studies have documented an increased risk for embolic events, including ischemic stroke, in the first 30 days after a COVID-19 infection, with a >2-fold greater risk compared with people without COVID-19 (*6*,*7*). Several studies have found the risk for ischemic stroke is higher in those with severe acute illness (*8*,*9*). Among children, who have fewer strokes and thromboembolic events, 2 studies found an increased risk for stroke after COVID-19 (*10*,*11*). Although the mechanisms remain under investigation, the hypothesized pathophysiology that leads to increased stroke and thromboembolic events among patients with COVID-19 include endothelial cell damage (*12*,*13*), a viral-triggered exaggerated immune response and cytokine storm (*14*), and persistent microthrombi formation and fibrin amyloid microclots (*15*,*16*).

Limited information exists on stroke and thrombotic events in the postillness period beyond 30 days after COVID-19 infection. Many patients with risk factors for stroke, such as hypertension, high cholesterol, and smoking, might recover from COVID-19 but experience an elevated risk for thrombotic events beyond 30 days. In addition, whether the risk for stroke and thrombotic events in the months after SARS-CoV-2 infections is similar to that for other respiratory viruses (e.g., influenza) is unknown (*17*,*18*). Further, previous studies have focused on earlier SARS-CoV-2 variants (*19*,*20*), some of which were conducted before recommendations for thromboprophylaxis during hospitalization for COVID-19 (*21*), widespread use of COVID-19 treatments, or COVID-19 vaccination (*19*,*22*). Determining the incidence of stroke and thrombotic events in patients with COVID-19 or acute respiratory illnesses (ARI) in the 31–365 days after illness could help clarify long-term risk among patients with COVID-19 and point to possible interventions for prevention. We investigated incidence of thromboembolic events and stroke in the 31–365 days after COVID-19 diagnosis, both overall and by patient hospitalization status.

## Methods

We used data from the National Patient-Centered Clinical Research Network (PCORnet), a national research network containing comprehensive electronic health record data from healthcare systems across the United States (*23*). We examined the overall incidence of thromboembolic events and stroke, as well as the specific incidence of ischemic stroke, deep vein thrombosis (DVT), hemorrhagic stroke, transient ischemic attack (TIA), and cerebral venous sinus thrombosis (CVST), in the 31–365 days after COVID-19 diagnosis, overall and by patient hospitalization status, for the period April 1, 2022–April 30, 2023. We then used the incidences after an acute respiratory illness (ARI) in the same period as a comparison group. Because of a nonbillable diagnosis code being mistakenly used in the initial data pull, pulmonary embolism (PE) was not included in the analysis. Therefore, a patient with only a PE and no DVT diagnosed in the electronic health record (EHR) would not have been included in this analysis.

PCORnet provides data infrastructure to support distributed research across participating healthcare systems (*24*). PCORnet uses a Common Data Model to enable data interoperability and centralized querying of longitudinal EHR data by using modular statistical programs. Queries were performed at each participating healthcare system by using patient-level EHR data; results were transmitted to investigators in aggregated tabular format. Patient-level data were stored behind institutional firewalls. 

This activity was included in a larger surveillance program funded through a cooperative agreement by the Centers for Disease Control and Prevention (CDC) and was deemed exempt from review under the public health surveillance provision of the Common Rule by the Harvard Pilgrim Health Care institutional review board. The design and analysis adhere to the Strengthening the Reporting of Observational Studies in Epidemiology reporting guidelines (*25*). This activity was reviewed by CDC, deemed not research, and was conducted consistent with applicable federal law and CDC policy (See e.g., 45 C.F.R. part 46.102(l)(2), 21 C.F.R. part 56; 42 U.S.C. §241(d); 5 U.S.C. §552a; 44 U.S.C. §3501 et seq).

We selected the period April 1, 2022–April 30, 2023, to account for potential temporal confounding caused by changing treatment and prevention availability and changing predominant SARS-CoV-2 variants. We included patients who were >5 years of age; had COVID-19 or other ARI during April 1, 2022–April 30, 2023; had no evidence of pregnancy in the prior year; and had evidence of an encounter in the healthcare system in the 30–540 days before the COVID-19 or ARI diagnosis. An encounter with the healthcare system from 30–540 days prior was required to attempt to capture patient medical history, including prior strokes or thrombotic events. We stratified data by age, sex, race (American Indian or Alaska Native, Asian or Native Hawaiian or Pacific Islander, Black or African American, multiple races or other, White, or missing), and ethnicity (Hispanic, non-Hispanic, unknown, other, or missing).

Patients with COVID-19 were identified by a positive antigen or PCR laboratory record with a positive, detected, or presumptive positive result; receipt of a COVID-19 medication (monoclonal antibodies, nirmatrelvir/ritonavir, molnupiravir, or remdesivir); or COVID-19 diagnostic codes U07.1 or U07.2 from the International Classification of Diseases, 10th Revision, Clinical Modification. Patients with ARI were identified by an ARI or influenza diagnostic code (Appendix Table 1), receipt of oseltamivir or baloxavir, and no COVID-19 diagnosis from 365 days prior through 14 days after ARI diagnosis. Patients diagnosed with ischemic stroke, DVT, hemorrhagic stroke, TIA, or CVST in the 18 months before COVID-19 or ARI diagnosis were excluded from the cohorts when measuring the respective outcomes to better capture incident cases rather than prevalence or recurrent cases (*26*,*27*).

Among COVID-19 and ARI patients, cohorts were created on the basis of hospitalization status, whether or not a stroke or thrombotic event occurred, and the postacute period of focus. We calculated the incidence of any stroke or thrombotic event and the disaggregated categories of ischemic stroke, DVT, hemorrhagic stroke, TIA, or CVST in the time intervals after COVID-19 or ARI diagnosis: 31–90 days, 91–180 days, 181–365 days, and 31–365 days. Results were stratified according to patient hospitalization status (hospitalized vs. nonhospitalized from 1 day before through 16 days after COVID-19 or ARI diagnosis to reflect the period of acute illness). We chose those time intervals to characterize the postacute phase of illness (31–90 days), to remain consistent with the definition of long COVID (having symptoms for >90 days after COVID-19 diagnosis) (*28*), and to determine when events are most likely to occur (e.g., 91–180 days vs. 181–365 days). We used χ^2^ testing to assess significant differences at p<0.05.

We calculated the incidence of any event per 10,000 patients. We calculated each ischemic stroke, DVT, hemorrhagic stroke, TIA, and CVST event among patients with COVID-19 or ARI and stratified by hospitalization status and time from acute COVID-19 or ARI diagnosis. Within each period, we calculated the 30-day incidence. We calculated incidence ratios and 95% CIs on the basis of the normal distribution to compare the risk for stroke or TIA after COVID-19 versus ARI. We indicated significance at p<0.05 by using *Z*-test to assess the differences of proportions.

## Results

A total of 1,132,355 patients were diagnosed with COVID-19 and 2,301,209 patients with ARI (Appendix Table 2) during the study period. A higher proportion of patients who had COVID-19 were in older age groups (p<0.0001 by χ^2^ test) and had more chronic conditions (p<0.0001 by χ^2^ test) compared with patients who had ARI. Among patients diagnosed with COVID-19, the most common age groups were 50–64 (25.8%) and >65 years (32.5%); 5–17 years (34.0%) was the largest age group among patients with ARI. There was a greater percentage of females in both the COVID-19 (60.4% vs. 39.6% male) and ARI (59.8% vs. 40.2% male) cohorts. The most common race among patients with COVID-19 and ARI was White (COVID-19, 70.7%; ARI, 69.3%), followed by Black or African American (COVID-19, 14.5%; ARI, 15.1%). Most patients were non-Hispanic (COVID-19, 73.6%; ARI, 71.1%). The most common conditions in both COVID-19 and ARI patients in the 18 months before diagnosis were hypertension (COVID-19, 38.6%; ARI, 23.2%), hyperlipidemia (COVID-19, 30.2%; ARI, 17.4%), and diabetes (COVID-19, 16.8%; ARI, 10.4%) (Appendix Table 2).

A total of 17,606 patients with COVID-19 and 21,871 patients with ARI experienced an event (ischemic stroke, DVT, hemorrhagic stroke, TIA, or CVST) in the 31–365 days after COVID-19 or ARI diagnosis (Appendix Table 3). Adults >50 years of age were most of the patients with COVID-19 (88.2%). There was no significant difference between hospitalized (88.4%) and nonhospitalized (88.1%) COVID-19 patients >50 years of age (p = 0.63). Adults >50 years of age were most of the patients with ARI (83.5%), and this age group also represented most hospitalized patients (85.4%) (p<0.0001). Combining hospitalized and nonhospitalized patients, patients >50 years of age made up a greater percentage of COVID-19 patients (88.2%) than ARI patients (83.5%) in the same age group (p<0.0001).

Among both COVID-19 and ARI hospitalized patients who experienced an event in the 31–365 days after acute illness, underlying hypertension (COVID-19, 82.1%; ARI, 81.8%), hyperlipidemia (COVID-19, 60.0%; ARI, 60.1%), diabetes (COVID-19, 45.6%; ARI, 43.2%), coronary artery disease (COVID-19, 41.6%; ARI, 42.1%), and chronic kidney disease (COVID-19, 46.3%; ARI, 41.7%) were all more common than among nonhospitalized patients (p<0.0001) (Appendix Table 3). When comparing all COVID-19 patients who experienced an event in the 31–365 days after acute illness to all ARI patients who experienced a stroke or thrombotic event within 31–365 days after acute illness, a history of chronic kidney disease (p<0.0001), hyperlipidemia (p = 0.0019), and alcohol abuse (p = 0.0103) were statistically more common among COVID-19 patients. We found no significant difference between the percentage of COVID-19 and ARI patients with a history of coronary artery disease (p = 0.6835), diabetes (p = 0.9171), or hypertension (p = 0.2757).

Among COVID-19 patients, hospitalized patients were also more likely to have received anticoagulants in the prior 18 months (16.8%) than were nonhospitalized patients (10.7%) (p<0.0001). Among ARI patients, hospitalized patients were more likely to have received anticoagulants (16.4%) than were nonhospitalized patients (10.0%) (p<0.0001). When comparing all COVID-19 patients to all ARI patients, more COVID-19 patients had received antiplatelet medications in the 18 months prior (COVID-19, 9.1%; ARI, 8.1%) (p = 0.0009), but there was no statistical difference in the percentage of patients previously having received anticoagulants (COVID-19, 11.9%; ARI, 11.4%) (p = 0.124).

Overall, incidence of all events decreased as time increased after acute illness: 31–90 days, 20 events/10,000 COVID-19 patients and 13 events/10,000 ARI patients; 91–180 days, 19 events/10,000 COVID-19 patients and 12 events/10,000 ARI patients; 181–365 days, 16 events/10,000 COVID-19 patients and 10 events/10,000 ARI patients (Figure; Appendix Table 4). For all time intervals, incidence of all events was higher among patients who were hospitalized than patients who were nonhospitalized, and higher among patients with COVID-19 than patients with ARI. DVT and ischemic stroke were the most common events diagnosed in the year after acute illness for both COVID-19 (4 DVT/10,000 patients and 6 ischemic strokes/10,000 patients) and ARI (3 DVT/10,000 patients and 3 ischemic strokes/10,000 patients) (Appendix Table 2). DVT and ischemic stroke incidence was also higher among patients hospitalized with COVID-19 versus patients who were nonhospitalized and patients diagnosed with ARI; rates for those events decreased farther out from the acute illness (Appendix Table 4).

**Figure:**
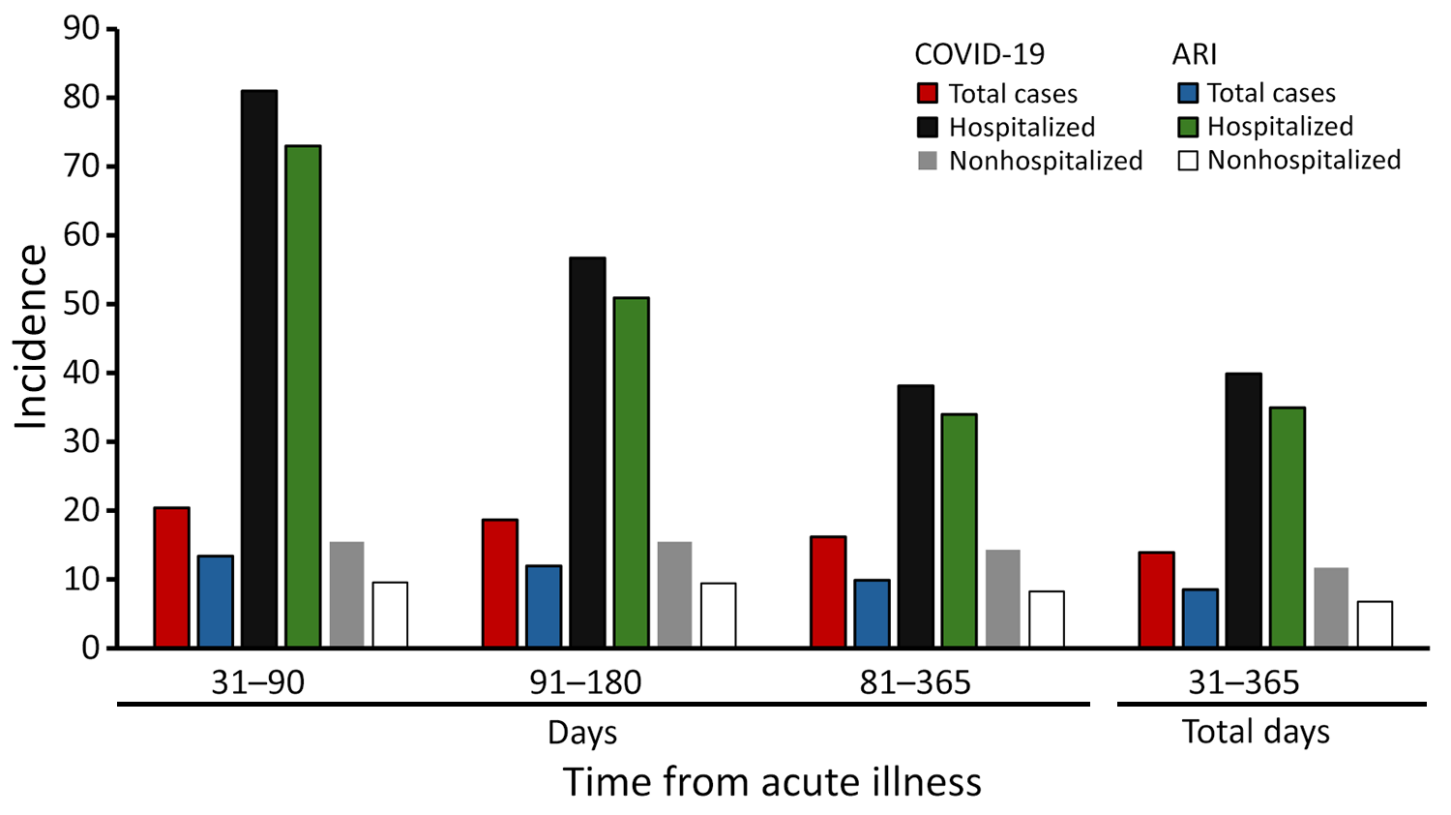
Thirty-day incidence of all events per 10,000 patients by acute illness, hospitalization status, and time from acute illness in a study of thrombotic events and stroke in the year after COVID-19 or other acute respiratory infection. We defined events as cerebral venous sinus thrombosis, deep vein thrombosis, hemorrhagic stroke, ischemic stroke, or transient ischemic attack.

The unadjusted risk for any event was higher among patients with COVID-19 compared with patients with ARI regardless of hospitalization status or time interval after acute illness (hospitalized patient incidence ratio 1.11 for 31–90 days and 91–180 days, 1.12 for 181–365 days; nonhospitalized patient incidence ratio 1.62 for 31–90 days, 1.64 for 91–180 days, 1.73 for 181–365 days) (p<0.0001) (Table). The unadjusted risk for ischemic stroke was higher among patients with COVID-19 compared with patients with ARI in every time interval and among both hospitalized and nonhospitalized patients (hospitalized patient incidence ratio 1.13 for 31–90 days, 1.26 for 91–180 days, 1.12 for 181–365 days; nonhospitalized patient incidence ratio 1.65 for 31–90 days, 1.68 for 91–180 days, 1.77 for 181–365 days) (p<0.0001). Hospitalized patients with COVID-19 had a 14% increased crude risk for any event measured in the 31–365 days after acute illness compared with patients with ARI (p<0.0001) (Table). Although the overall incidence of all events for nonhospitalized patients compared with hospitalized patients was lower for both COVID-19 and ARI (Appendix Table 3), nonhospitalized patients with COVID-19 had a 73% increased crude risk for any event in the year after acute illness compared with nonhospitalized patients with ARI (p<0.0001) (Table). Among hospitalized patients, when comparing risk by specific outcomes, there was an increased risk for DVT (11%, p<0.0001), ischemic stroke (20%, p<0.0001), and TIA (27%, p<0.0001) among COVID-19 versus ARI patients but no significant difference for CVST or hemorrhagic stroke.

**Table:** Unadjusted incidence ratios of stroke and thrombotic events for patients with COVID-19 compared with ARI by hospitalization status and days from acute illness*

All events	Unadjusted ratio of COVID-19 to ARI (95% CI)		
	All patients	Hospitalized	Nonhospitalized
All events 31–90 d and no record in 18 mo prior	**1.52 (1.48–1.56)**	**1.11 (1.04–1.18)**	**1.62 (1.58–1.67)**
Ischemic stroke	**1.55 (1.49–1.61)**	**1.13 (1.02–1.25)**	**1.65 (1.58–1.73)**
Deep vein thrombosis	**1.51 (1.44–1.57)**	**1.12 (1.01–1.23)**	**1.62 (1.53–1.7)**
Hemorrhagic stroke	**1.47 (1.36–1.58)**	0.99 (0.81–1.16)	**1.71 (1.57–1.85)**
Transient ischemic attack	**1.56 (1.47–1.65)**	1.2 (0.99–1.41)	**1.6 (1.51–1.7)**
Cerebral venous sinus thrombosis	1.09 (0.72–1.45)	0.92 (0.31–1.53)	1.09 (0.63–1.54)
All events 91–180 d and no record in 18 mo prior	**1.55 (1.52–1.59)**	**1.11 (1.04–1.18)**	**1.64 (1.61–1.68)**
Ischemic stroke	**1.63 (1.58–1.68)**	**1.26 (1.16–1.37)**	**1.68 (1.62–1.74)**
Deep vein thrombosis	**1.5 (1.44–1.56)**	1.08 (0.97–1.19)	**1.6 (1.53–1.66)**
Hemorrhagic stroke	**1.43 (1.34–1.53)**	0.91 (0.73–1.09)	**1.62 (1.51–1.73)**
Transient ischemic attack	**1.56 (1.49–1.64)**	1.15 (0.94–1.35)	**1.61 (1.53–1.69)**
Cerebral venous sinus thrombosis	**1.35 (1.03–1.66)**	0.87 (0.24–1.49)	**1.49 (1.12–1.85)**
All events 181–365 d and no record in 18 mo prior	**1.64 (1.62–1.66)**	**1.12 (1.07–1.18)**	**1.73 (1.71–1.76)**
Ischemic stroke	**1.68 (1.64–1.72)**	**1.16 (1.07–1.25)**	**1.77 (1.73–1.82)**
Deep vein thrombosis	**1.62 (1.57–1.66)**	1.08 (0.98–1.19)	**1.73 (1.68–1.78)**
Hemorrhagic stroke	**1.58 (1.51–1.65)**	0.92 (0.77–1.08)	**1.79 (1.7–1.87)**
Transient ischemic attack	**1.69 (1.63–1.74)**	**1.34 (1.18–1.49)**	**1.72 (1.66–1.77)**
Cerebral venous sinus thrombosis	**1.38 (1.16–1.6)**	1.13 (0.6–1.66)	**1.4 (1.15–1.65)**
All events 31–365 d and no record in 18 mo prior	**1.63 (1.61–1.65)**	**1.14 (1.1–1.18)**	**1.73 (1.71–1.76)**
Ischemic stroke	**1.67 (1.64–1.7)**	**1.2 (1.14–1.27)**	**1.76 (1.73–1.8)**
Deep vein thrombosis	**1.59 (1.55–1.62)**	**1.11 (1.04–1.18)**	**1.7 (1.66–1.74)**
Hemorrhagic stroke	**1.56 (1.51–1.62)**	0.98 (0.87–1.09)	**1.77 (1.7–1.84)**
Transient ischemic attack	**1.42 (1.37–1.46)**	**1.27 (1.16–1.39)**	**1.42 (1.37–1.46)**
Cerebral venous sinus thrombosis	**1.36 (1.18–1.55)**	0.95 (0.53–1.37)	**1.44 (1.23–1.65)**

*Bold indicates statistically significant ratios. ARI, acute respiratory illness

## Discussion

Among patients with COVID-19 and ARI illness, the greatest risk for incident thrombotic events, including stroke, occurred within 31–90 days after acute illness. Incident thrombotic events continued up to a year after acute illness. Overall, hospitalized patients had the highest incidence of postillness events, and patients with COVID-19 had higher incidence of thrombotic events compared with patients with ARI, regardless of hospitalization status.

Although the incidence of all events decreased as the time from acute illness increased, overall, increased awareness of risk for such events in COVID-19 patients is justified well past 1 month after acute infection. It is critical that healthcare providers maintain awareness of the risk for stroke and thromboembolic events in patients even after recovery from acute COVID-19 infection and monitor those at risk, particularly patients with known risk factors such as hypertension, high cholesterol, diabetes, obesity, smoking, sedentary lifestyle, or previous venous thromboembolisms. Current clinical guidelines recommend thromboprophylaxis during hospitalization for many adults hospitalized with COVID-19 but not after discharge or for those treated in outpatient settings (*29*). A small trial in 2021 showed improved clinical outcomes with thromboprophylaxis after hospital discharge in high-risk patients (*30*), whereas another trial published in 2023 showed no significant difference in outcomes between COVID-19 outpatients who did and did not receive thromboprophylaxis and was stopped early because of low thromboembolic incidence rates (*31*). The risk of bleeding from anticoagulation would ideally be balanced with thromboembolic event prevention. However, this study was not designed to determine the usefulness of thromboprophylaxis or other risk modifications after hospital discharge or in outpatient settings; further study is needed to inform that determination.

Rates for all events we tracked—DVT, hemorrhagic stroke, ischemic stroke, and TIA—were higher among hospitalized than nonhospitalized patients with COVID-19 across all time intervals. The higher rates likely reflect the higher hospitalization rates among patients at greater risk for severe COVID-19, such as those >50 years of age or with multiple comorbidities. In addition, the higher rates for all events suggest an increased risk for stroke and thromboembolic events associated with more severe acute illness (*8*,*9*,*32*,*33*). Determining the biological mechanisms driving the increased incidence of stroke and thromboembolic events after COVID-19 infection could help identify patients at higher risk and inform prevention strategies. Current thromboprophylaxis recommendations in patients with COVID-19 are limited to select hospitalized patients (*34*–*37*).

Of note, the risk ratios for all events in COVID-19 versus ARI patients were higher among the nonhospitalized group in this analysis, with a risk ratio of 1.73 (95% CI 1.71–1.76) for 31–365 days among nonhospitalized patients versus 1.14 (95% CI 1.10–1.18) in hospitalized patients. Many earlier studies focused on the initial phases of the COVID-19 pandemic, primarily during the pre-Delta and Delta variant periods (*19*,*20*). In contrast, this study provides more recent data from the Omicron-dominant period, characterized by high population immunity because of extensive vaccination and prior infections. Those updated findings could provide valuable insights for future studies and enhance early recognition and effective management of DVT and stroke, while informing the long-term cardiovascular consequences of COVID-19.

This study underscores the importance of COVID-19 vaccination and other prevention and treatment efforts to reduce risk for severe illness and subsequent adverse outcomes and conditions (*38*). In addition, given the higher risk for post-COVID conditions with more severe COVID-19 acute illness (*39*,*40*), our data provide yet another reason to increase efforts targeted at prevention and improved management of chronic conditions that increase the risk for severe COVID-19, stroke, and thrombotic complications. Comprehensive chronic disease management, combined with COVID-19 and ARI prevention strategies, can help reduce the incidence of postillness DVT and stroke, ultimately benefiting those most vulnerable to complications. Patient education is also crucial, particularly an emphasis on the benefits of vaccinations for those with underlying risk factors or comorbidities.

The first limitation of this study is that this analysis does not include biological measurements or pathophysiology information to assign direct causation of SARS-CoV-2 infection to stroke incidence. Second, because of the aggregated data for this analysis, we could not adjust for patient level potential confounders. Compared with patients with ARI, COVID-19 patients were in older age groups and had more chronic conditions; adjusting for those potential confounders might attenuate the differences across the groups. Although unadjusted risk ratios for all events in COVID-19 versus ARI patients were higher among the nonhospitalized group, those are relative risks, and the difference in risks might be influenced by selection bias. Hospitalized patients are more likely to get virus-specific testing to guide therapy, and patients with mild illness might have visited a provider for a diagnosis, but those with mild illness are less likely to get tested with a virus-specific test compared with those with moderate symptoms (*41*,*42*). Therefore, it is possible that hospitalized patients were more likely to be correctly categorized between COVID-19 and other ARIs, compared with those who were only seen as outpatients and might not have had definitive testing. The nonhospitalized ARI cohort was younger and healthier than the nonhospitalized COVID-19 cohort, suggesting a lower baseline risk of thromboembolism and stroke. That age difference might have led to an overestimation of the effect of COVID-19 on the thromboembolic and stroke risk. Third, only patients who had access to and sought clinical care for their acute COVID-19 or ARI illness or subsequent event were included, likely leading to the exclusion of some people with mild or asymptomatic infections and persons who never received laboratory testing for COVID-19 or ARI. Fourth, persons with mild DVT or TIA who did not seek care were also not included. Fifth, because of a coding error identified that could not be corrected after the data was received, this analysis also did not include PE as an outcome. Patients with DVT who only saw a provider after a PE developed might have been diagnosed with PE alone, leading to potential undercount of DVT cases. Sixth, excluding persons with a prior history of events (DVT, ischemic stroke, hemorrhagic stroke, PE, or TIA) limits the representativeness of the analytic sample and therefore limits the generalizability of our findings to the US population. Finally, the study period (April 1, 2022–April 30, 2023) includes various Omicron sublineages but was not designed to align with specific sublineage periods.

Future analyses with line level data available could control for patients’ chronic conditions to better identify thrombotic events attributable to viral infection. Separating out specific pathogens, such as influenza, from other ARI could also help quantify the risks of specific viruses.

In conclusion, this study identified a possible elevated risk for thrombotic events, including stroke, up to a year after COVID-19, especially among patients hospitalized with COVID-19. This risk appears to remain higher for patients with COVID-19 than for those with ARI. Future multivariate analysis with adjustments for demographic and medical differences is needed. Continued surveillance and epidemiologic studies are essential to monitor these long-term risks and assess mitigation strategies. This study also underscores the importance of stroke awareness. By recognizing stroke signs and symptoms, such as by using the FAST acronym (*43*), patients and providers can help ensure timely intervention, potentially improving recovery outcomes and reducing disability and mortality (*44*).

Appendix Additional information about thrombotic events and stroke in the year after COVID-19 or other acute respiratory infection.

## References

[1] Bahouth MN, Venkatesan A. Acute viral illnesses and ischemic stroke: pathophysiological considerations in the era of the COVID-19 pandemic. Stroke. 2021;52:1885–94. 10.1161/STROKEAHA.120.030630 33794653 PMC8078120

[2] Nguyen TQ, Vlasenko D, Shetty AN, Reid CM, Clothier HJ, Buttery JP. Laboratory-confirmed respiratory viral infection triggers for acute myocardial infarction and stroke: Systematic review protocol. PLoS One. 2024;19:e0302748. 10.1371/journal.pone.0302748 38985724 PMC11236192

[3] Smeeth L, Thomas SL, Hall AJ, Hubbard R, Farrington P, Vallance P. Risk of myocardial infarction and stroke after acute infection or vaccination. N Engl J Med. 2004;351:2611–8. 10.1056/NEJMoa041747 15602021

[4] Babkina AS, Pisarev MV, Grechko AV, Golubev AM. Arterial thrombosis in acute respiratory infections: an underestimated but clinically relevant problem. J Clin Med. 2024;13:6007. 10.3390/jcm13196007 39408067 PMC11477565

[5] Ward A, Sarraju A, Lee D, Bhasin K, Gad S, Beetel R, et al. COVID-19 is associated with higher risk of venous thrombosis, but not arterial thrombosis, compared with influenza: Insights from a large US cohort. PLoS One. 2022;17:e0261786. 10.1371/journal.pone.0261786 35020742 PMC8754296

[6] Katsoularis I, Fonseca-Rodríguez O, Farrington P, Lindmark K, Fors Connolly AM. Risk of acute myocardial infarction and ischaemic stroke following COVID-19 in Sweden: a self-controlled case series and matched cohort study. Lancet. 2021;398:599–607. 10.1016/S0140-6736(21)00896-5 34332652 PMC8321431

[7] Tu TM, Seet CYH, Koh JS, Tham CH, Chiew HJ, De Leon JA, et al. Acute ischemic stroke during the convalescent phase of asymptomatic COVID-2019 infection in men. JAMA Netw Open. 2021;4:e217498. 10.1001/jamanetworkopen.2021.7498 33885771 PMC8063067

[8] Siepmann T, Sedghi A, Simon E, Winzer S, Barlinn J, de With K, et al. Increased risk of acute stroke among patients with severe COVID-19: a multicenter study and meta-analysis. Eur J Neurol. 2021;28:238–47. 10.1111/ene.14535 32920964

[9] Nannoni S, de Groot R, Bell S, Markus HS. Stroke in COVID-19: A systematic review and meta-analysis. Int J Stroke. 2021;16:137–49. 10.1177/1747493020972922 33103610 PMC7859578

[10] Kompaniyets L, Bull-Otterson L, Boehmer TK, Baca S, Alvarez P, Hong K, et al. Post-COVID-19 symptoms and conditions among children and adolescents—United States, March 1, 2020–January 31, 2022. MMWR Morb Mortal Wkly Rep. 2022;71:993–9. 10.15585/mmwr.mm7131a3 35925799 PMC9368731

[11] Vielleux MJ, Swartwood S, Nguyen D, James KE, Barbeau B, Bonkowsky JL. SARS-CoV-2 infection and increased risk for pediatric stroke. Pediatr Neurol. 2023;142:89–94. 10.1016/j.pediatrneurol.2022.10.003 36418211 PMC9675636

[12] Koyama AK, Imperatore G, Rolka DB, Lundeen E, Rutkowski RE, Jackson SL, et al. Risk of cardiovascular disease after COVID-19 diagnosis among adults with and without diabetes. J Am Heart Assoc. 2023;12:e029696. 10.1161/JAHA.123.029696 37382101 PMC10356070

[13] Ackermann M, Mentzer SJ, Kolb M, Jonigk D. Inflammation and intussusceptive angiogenesis in COVID-19: everything in and out of flow. Eur Respir J. 2020;56:2003147. 10.1183/13993003.03147-2020 33008942 PMC7530910

[14] Silva Andrade B, Siqueira S, de Assis Soares WR, de Souza Rangel F, Santos NO, Dos Santos Freitas A, et al. Long-COVID and post-COVID health complications: an up-to-date review on clinical conditions and their possible molecular mechanisms. Viruses. 2021;13:700. 10.3390/v13040700 33919537 PMC8072585

[15] Ranucci M, Baryshnikova E, Anguissola M, Pugliese S, Falco M, Menicanti L. The long term residual effects of COVID-associated coagulopathy. Int J Mol Sci. 2023;24:5514. 10.3390/ijms24065514 36982589 PMC10049638

[16] Kell DB, Laubscher GJ, Pretorius E. A central role for amyloid fibrin microclots in long COVID/PASC: origins and therapeutic implications. Biochem J. 2022;479:537–59. 10.1042/BCJ20220016 35195253 PMC8883497

[17] Kulick ER, Alvord T, Canning M, Elkind MSV, Chang BP, Boehme AK. Risk of stroke and myocardial infarction after influenza-like illness in New York State. BMC Public Health. 2021;21:864. 10.1186/s12889-021-10916-4 33952233 PMC8097921

[18] Boehme AK, Luna J, Kulick ER, Kamel H, Elkind MSV. Influenza-like illness as a trigger for ischemic stroke. Ann Clin Transl Neurol. 2018;5:456–63. 10.1002/acn3.545 29687022 PMC5899905

[19] Xie Y, Xu E, Bowe B, Al-Aly Z. Long-term cardiovascular outcomes of COVID-19. Nat Med. 2022;28:583–90. 10.1038/s41591-022-01689-3 35132265 PMC8938267

[20] Yang Q, Chang A, Tong X, Jackson SL, Merritt RK. Long-term cardiovascular disease outcomes in non-hospitalized medicare beneficiaries diagnosed with COVID-19: Population-based matched cohort study. PLoS One. 2024;19:e0302593. 10.1371/journal.pone.0302593 38743728 PMC11093379

[21] Lawler PR, Goligher EC, Berger JS, Neal MD, McVerry BJ, Nicolau JC, et al.; ATTACC Investigators; ACTIV-4a Investigators; REMAP-CAP Investigators. ACTIV-4a Investigators; REMAP-CAP Investigators. Therapeutic anticoagulation with heparin in noncritically ill patients with COVID-19. N Engl J Med. 2021;385:790–802. 10.1056/NEJMoa2105911 34351721 PMC8362594

[22] Yang Q, Tong X, George MG, Chang A, Merritt RK. COVID-19 and risk of acute ischemic stroke among medicare beneficiaries aged 65 years or older: self-controlled case series study. Neurology. 2022;98:e778–89. 10.1212/WNL.0000000000013184 35115387 PMC8935393

[23] Ghildayal N, Nagavedu K, Wiltz JL, Back S, Boehmer TK, Draper C, et al. Public health surveillance in electronic health records: lessons from PCORnet. Prev Chronic Dis. 2024;21:E51. 10.5888/pcd21.230417 38991533 PMC11262136

[24] Forrest CB, McTigue KM, Hernandez AF, Cohen LW, Cruz H, Haynes K, et al. PCORnet® 2020: current state, accomplishments, and future directions. J Clin Epidemiol. 2021;129:60–7. 10.1016/j.jclinepi.2020.09.036 33002635 PMC7521354

[25] von Elm E, Altman DG, Egger M, Pocock SJ, Gøtzsche PC, Vandenbroucke JP; STROBE Initiative. The Strengthening the Reporting of Observational Studies in Epidemiology (STROBE) statement: guidelines for reporting observational studies. J Clin Epidemiol. 2008;61:344–9. 10.1016/j.jclinepi.2007.11.008 18313558

[26] Kolmos M, Christoffersen L, Kruuse C. Recurrent ischemic stroke—a systematic review and meta-analysis. J Stroke Cerebrovasc Dis. 2021;30:105935. 10.1016/j.jstrokecerebrovasdis.2021.105935 34153594

[27] Verburgt E, Hilkens NA, Ekker MS, Schellekens MMI, Boot EM, Immens MHM, et al. Short-term and long-term risk of recurrent vascular event by cause after ischemic stroke in young adults. JAMA Netw Open. 2024;7:e240054. 10.1001/jamanetworkopen.2024.0054 38376841 PMC10879951

[28] Committee on Examining the Working Definition for Long COVID, Board on Health Sciences Policy, Board on Global Health, Health and Medicine Division, National Academies of Sciences, Engineering, and Medicine. A long COVID definition: a chronic, systemic disease state with profound consequences. Fineberg HV, Brown L, Worku T, Goldowitz I, editors. Washington: National Academies Press; 2024. 39110819

[29] Cuker A. COVID-19: hypercoagulability. 2024 [cited 2024 Oct 31]. https://www.uptodate.com/contents/covid-19-hypercoagulability#H3177214199

[30] Ramacciotti E, Barile Agati L, Calderaro D, Aguiar VCR, Spyropoulos AC, de Oliveira CCC, et al.; MICHELLE investigators. Rivaroxaban versus no anticoagulation for post-discharge thromboprophylaxis after hospitalisation for COVID-19 (MICHELLE): an open-label, multicentre, randomised, controlled trial. Lancet. 2022;399:50–9. 10.1016/S0140-6736(21)02392-8 34921756 PMC8673881

[31] Connors JM, Brooks MM, Sciurba FC, Krishnan JA, Bledsoe JR, Kindzelski A, et al.; ACTIV-4B Investigators. Effect of antithrombotic therapy on clinical outcomes in outpatients with clinically stable symptomatic COVID-19: the ACTIV-4B randomized clinical trial. JAMA. 2021;326:1703–12. 10.1001/jama.2021.17272 34633405 PMC8506296

[32] Luo W, Liu X, Bao K, Huang C. Ischemic stroke associated with COVID-19: a systematic review and meta-analysis. J Neurol. 2022;269:1731–40. 10.1007/s00415-021-10837-7 34652503 PMC8517946

[33] Rothstein A, Oldridge O, Schwennesen H, Do D, Cucchiara BL. Acute cerebrovascular events in hospitalized COVID-19 patients. Stroke. 2020;51:e219–22. 10.1161/STROKEAHA.120.030995 32684145 PMC7386677

[34] Centers for Disease Control and Prevention. COVID-19 clinical treatment clinical care for outpatients. 2024 [cited 2024 Nov 1]. https://www.cdc.gov/covid/hcp/clinical-care/outpatient-treatment.html

[35] Centers for Disease Control and Prevention. Benefits of getting vaccinated. 2024 [cited 2024 Nov 1]. https://www.cdc.gov/covid/vaccines/benefits.html

[36] Mercadé-Besora N, Li X, Kolde R, Trinh NT, Sanchez-Santos MT, Man WY, et al. The role of COVID-19 vaccines in preventing post-COVID-19 thromboembolic and cardiovascular complications. Heart. 2024;110:635–43. 10.1136/heartjnl-2023-323483 38471729 PMC11041555

[37] Payne AB, Novosad S, Wiegand RE, Najdowski M, Gomes DJ, Wallace M, et al. Effectiveness of bivalent mRNA COVID-19 vaccines in preventing COVID-19-related thromboembolic events among medicare enrollees aged >65 years and those with end stage renal disease—United States, September 2022–March 2023. MMWR Morb Mortal Wkly Rep. 2024;73:16–23. 10.15585/mmwr.mm7301a4 38206877 PMC10794061

[38] Romero-Rodríguez E, Pérula-de Torres LÁ, Castro-Jiménez R, González-Lama J, Jiménez-García C, González-Bernal JJ, et al. Hospital admission and vaccination as predictive factors of long COVID-19 symptoms. Front Med (Lausanne). 2022;9:1016013. 10.3389/fmed.2022.1016013 36438042 PMC9691755

[39] Hastie CE, Lowe DJ, McAuley A, Winter AJ, Mills NL, Black C, et al. Outcomes among confirmed cases and a matched comparison group in the Long-COVID in Scotland study. Nat Commun. 2022;13:5663. 10.1038/s41467-022-33415-5 36224173 PMC9556711

[40] Centers for Disease Control and Prevention. Long COVID basics. 2025 [cited 2025 Apr 29]. https://www.cdc.gov/covid/long-term-effects/index.html

[41] Heikkinen T, Järvinen A. The common cold. Lancet. 2003;361:51–9. 10.1016/S0140-6736(03)12162-9 12517470 PMC7112468

[42] Gonzales R, Malone DC, Maselli JH, Sande MA. Excessive antibiotic use for acute respiratory infections in the United States. Clin Infect Dis. 2001;33:757–62. 10.1086/322627 11512079

[43] Centers for Disease Control and Prevention. Signs and symptoms of stroke [cited 2024 Oct 24]. https://www.cdc.gov/stroke/signs-symptoms/index.html

[44] Chen X, Zhao X, Xu F, Guo M, Yang Y, Zhong L, et al. A systematic review and meta-analysis comparing FAST and BEFAST in acute stroke patients. Front Neurol. 2022;12:765069. 10.3389/fneur.2021.765069 35153975 PMC8837419

