# Supplementary material for: Thrombotic Events and Stroke in the Year After COVID-19 or Other Acute Respiratory Infection
Source: Emerg Infect Dis. 2025 Dec;31(Suppl 2):S3–S10. doi: 10.3201/eid3114.250630 (PMC12829552; doi:10.3201/eid3114.250630)
Supplement: Appendix — Additional information about thrombotic events and stroke in the year after COVID-19 or other acute respiratory infection. [file 25-0630-Techapp-s1.pdf]

Article DOI: https://doi.org/10.3201/eid3114.250630

EID cannot ensure accessibility for supplementary materials supplied by authors. Readers who have difficulty accessing supplementary content should contact the authors for assistance.

Thrombotic Events and Stroke in the Year After COVID-19 or Other Acute Respiratory Infection

Appendix Table 1. List of International Classification of Diseases, 10th Revision, Clinical Modification (ICD-10-CM) used for identifying patients with influenza or other acute respiratory infection.

ICD-10 Code	Description
J09	Influenza due to certain identified influenza viruses
J09.X	Influenza due to identified novel influenza A virus
J09.X1	Influenza due to identified novel influenza A virus with pneumonia
J09.X2	Influenza due to identified novel influenza A virus with other respiratory manifestations
J09.X3	Influenza due to identified novel influenza A virus with gastrointestinal manifestations
J09.X9	Influenza due to identified novel influenza A virus with other manifestations
J10.1	Influenza due to other identified influenza virus with other respiratory manifestations
J10.2	Influenza due to other identified influenza virus with gastrointestinal manifestations
J10.81	Influenza due to other identified influenza virus with encephalopathy
J10.82	Influenza due to other identified influenza virus with myocarditis
J10.83	Influenza due to other identified influenza virus with otitis media
J10.89	Influenza due to other identified influenza virus with other manifestations
J11.1	Influenza due to unidentified influenza virus with other respiratory manifestations
J11.2	Influenza due to unidentified influenza virus with gastrointestinal manifestations
J11.8	Influenza due to unidentified influenza virus with other manifestations
J11.81	Influenza due to unidentified influenza virus with encephalopathy
J11.82	Influenza due to unidentified influenza virus with myocarditis
J11.83	Influenza due to unidentified influenza virus with otitis media
J11.89	Influenza due to unidentified influenza virus with other manifestations
J09.01	Influenza due to identified avian influenza virus with respiratory manifestations
J09.010	Influenza due to identified avian influenza virus with identified avian influenza pneumonia
J09.018	Influenza due to identified avian influenza virus with other specified type of pneumonia
J09.019	Influenza due to identified avian influenza virus with unspecified type of pneumonia
J09.02	Influenza due to identified avian influenza virus with other respiratory manifestations
J09.03	Influenza due to identified avian influenza virus with gastrointestinal manifestations
J09.090	Influenza due to identified avian influenza virus with encephalopathy
J09.091	Influenza due to identified avian influenza virus with myocarditis
J09.092	Influenza due to identified avian influenza virus with otitis media
J09.098	Influenza due to identified avian influenza virus with other manifestations
J09.11	Influenza due to identified novel H1N1 influenza virus with respiratory manifestations
J09.110	Influenza due to identified novel H1N1 influenza virus with identified novel H1N1 influenza pneumonia
J09.118	Influenza due to identified novel H1N1 influenza virus with other specified type of pneumonia
J09.119	Influenza due to identified novel H1N1 influenza virus with unspecified type of pneumonia
J09.12	Influenza due to identified novel H1N1 influenza virus with other respiratory manifestations
J09.13	Influenza due to identified novel H1N1 influenza virus with gastrointestinal manifestations
J09.190	Influenza due to identified novel H1N1 influenza virus with encephalopathy
J09.191	Influenza due to identified novel H1N1 influenza virus with myocarditis
J09.192	Influenza due to identified novel H1N1 influenza virus with otitis media
J09.198	Influenza due to identified novel H1N1 influenza virus with other manifestations
J10	Influenza due to other identified influenza virus
J10.0	Influenza due to other identified influenza virus with pneumonia
J10.00	Influenza due to other identified influenza virus with unspecified type of pneumonia
J10.01	Influenza due to other identified influenza virus with the same other identified influenza virus pneumonia
J10.08	Influenza due to other identified influenza virus with other specified pneumonia
J10.8	Influenza with other manifestations, seasonal influenza virus identified
J11	Influenza, virus not identified
J11.0	Influenza due to unidentified influenza virus with pneumonia

ICD-10 Code	Description
J11.00	Influenza due to unidentified influenza virus with unspecified type of pneumonia
J11.08	Influenza due to unidentified influenza virus with specified pneumonia
J00	Acute nasopharyngitis (common cold)
J01*	Acute sinusitis
J02*	Acute pharyngitis
J03*	Acute tonsillitis
J04*	Acute laryngitis and tracheitis
J05*	Acute obstructive laryngitis and epiglottis
J06*	Acute upper respiratory infections of multiple or unspecified sites
J12	Viral pneumonia, not elsewhere classified
J12.0	Adenoviral pneumonia
J12.1	Respiratory syncytial virus pneumonia
J12.2	Parainfluenza virus pneumonia
J12.3	Human metapneumovirus pneumonia
J12.8	Other viral pneumonia
J12.81	Pneumonia due to SARS-associated coronavirus
J12.89	Other viral pneumonia, influenza bronchopneumonia
J12.9	Viral pneumonia, unspecified
J13	Strep pneumoniae pneumonia
J14	Hemophilus influenzae Pneumonia
J15	Bacterial pneumonia not elsewhere classified
J15.0	Klebsiella pneumoniae Pneumonia
J15.1	Pneumonia due to Pseudomonas
J15.2	Pneumonia due to staphylococcus
J15.20	Staphylococcus, unspecified
J15.211	MSSA Pneumonia
J15.212	MRSA Pneumonia
J15.29	Other Staphylococcus pneumonia
J15.3	Streptococcus, group B
J15.4	Pneumonia due to other Streptococcus
J15.5	Pneumonia due to *Escherichia coli*
J15.6	Other gram-negative bacteria
J15.7	Pneumonia due to Mycoplasma pneumoniae
J15.8	Pneumonia due to other specified bacteria
J15.9	Bacterial pneumonia, unspecified
J16	Pneumonia, other specified infectious organism
J16.0	Pneumonia due to Chlamydia
J16.8	Pneumonia due to other specified organism
J17	Pneumonia in diseases classified elsewhere
J18	Pneumonia, unspecified organism
J18.0	Pneumonia, unspecified organism
J18.1	Lobar pneumonia, unspecified organism
J18.2	Hypostatic pneumonia, unspecified organism
J18.8	Other pneumonia, unspecified organism
J18.9	Pneumonia, unspecified organism
J20	Acute bronchitis
J20.0	Acute bronchitis due to Mycoplasma pneumoniae
J20.1	Acute bronchitis due to Hemophilus influenzae
J20.2	Acute bronchitis due to streptococcus
J20.3	Acute bronchitis due to coxsackievirus
J20.4	Acute bronchitis due to parainfluenza virus
J20.5	Acute bronchitis due to respiratory syncytial virus
J20.6	Acute bronchitis due to rhinovirus
J20.7	Acute bronchitis due to echovirus
J20.8	Acute bronchitis due to other specified organisms
J20.9	Acute bronchitis, unspecified
J21	Acute bronchiolitis
J21.0	Acute bronchiolitis due to respiratory syncytial virus
j21.1	Acute bronchiolitis due to human metapneumovirus
J21.8	Acute bronchiolitis due to other specified organisms
J21.9	Acute bronchiolitis, unspecified
J22	Unspecified acute lower respiratory tract infection
J40	Bronchitis, not specified as acute or chronic
J80	Acute respiratory distress syndrome
J98.8	Other specified respiratory disorders
A22.1	Pulmonary anthrax
A37.01	Whooping cough: Bordetella pertussis, pneumonia
A37.11	Whooping cough: B. parapertussis, pneumonia
A37.81	Whooping cough: other Bordetella, pneumonia

ICD-10 Code	Description
A37.91	Whooping cough, unspecified species, pneumonia
A48.1	Legionnaire’s Disease
B25.0	Cytomegaloviral pneumonitis
B44.0	Invasive pulmonary aspergillosis
B97.4	Respiratory syncytial virus as the cause of diseases classified elsewhere
O98.5	Other viral diseases complicating pregnancy, childbirth and the puerperium

Appendix Table 2. Characteristics of patients diagnosed with COVID-19 or Acute Respiratory Infection (ARI) in PCORnet*, April 1, 2022 – April 30, 2023.

	All Patients					COVID-19					ARI				
	COVID-19		ARI			Hospitalized		Non-hospitalized			Hospitalized		Non-hospitalized		
Characteristics	N	%	N	%	p-value	N	%	N	%	p-value	N	%	N	%	p-value
Total	**1,132,355**		**2,301,209**			**80,209**		**1,070,125**			**134,392**		**2,198,472**		
**Demographics**															
**Age (years)**															
5–17	86,425	7.6	781,330	34.0	<0.0001	3,693	4.6	83,136	7.8	<0.0001	16,067	12.0	770,673	35.1	<0.0001
18–34	169,063	14.9	412,421	17.9		5,249	6.5	164,683	15.4		8,669	6.5	405,796	18.5	
35–49	216,986	19.2	359,660	15.6		7,680	9.6	210,792	19.7		12,135	9.0	350,296	15.9	
50–64	291,638	25.8	367,316	16.0		16,891	21.1	278,367	26.0		29,270	21.8	344,525	15.7	
≥65	368,243	32.5	380,482	16.5		46,696	58.2	333,147	31.1		68,251	50.8	327,182	14.9	
**Sex**															
Female	683,790	60.4	1,376,399	59.8	<0.0001	41,586	51.8	651,657	60.9	<0.0001	68,739	51.1	1,324,905	60.3	<0.0001
Male	448,306	39.6	924,498	40.2		38,615	48.1	418,217	39.1		65,638	48.8	873,267	39.7	
Unknown/Missing	259	0.0	312	0.0		8	0.0	251	0.0		15	0.0	300	0.0	
**Race**															
American Indian or Alaska Native	7,655	0.7	17,187	0.7	<0.0001	472	0.6	7,268	0.7	<0.0001	949	0.7	16,510	0.8	<0.0001
Asian or NHPI	39,565	3.5	65,535	2.8		1,772	2.2	38,193	3.6		3,204	2.4	63,015	2.9	
Black or African American	164,559	14.5	348,384	15.1		16,198	20.2	151,628	14.2		27,798	20.7	326,401	14.8	
Missing (No Information, Refuse to Answer, Unknown, Missing)	70,544	6.2	147,773	6.4		4,239	5.3	67,224	6.3		6,462	4.8	142,536	6.5	
Multiple Race or Other	49,687	4.4	127,411	5.5		3,841	4.8	46,643	4.4		6,855	5.1	122,207	5.6	
White	800,345	70.7	1,594,919	69.3		53,687	66.9	759,169	70.9		89,124	66.3	1,527,803	69.5	
**Hispanic Ethnicity**															
Yes	112,125	9.9	297,852	12.9	<0.0001	7,698	9.6	105,911	9.9	<0.0001	12,162	9.0	288,414	13.1	<0.0001
No	833,135	73.6	1,635,075	71.1		64,002	79.8	783,655	73.2		106,666	79.4	1,553,440	70.7	
Unknown, Other, or Missing	187,095	16.5	368,282	16.0		8,509	10.6	180,559	16.9		15,564	11.6	356,618	16.2	
**Past Medical History**															
**18 months before index event:**															
Acute COVID-19 Treatments	365,811	32.3	9748	0.4	<0.0001	19,714	24.6	355,947	33.3	<0.0001	1,534	1.1	8483	0.4	<0.0001
Alcohol Use Disorder	22,607	2.0	35,546	1.5	<0.0001	5,661	7.1	18,026	1.7	<0.0001	10,107	7.5	27,192	1.2	<0.0001
Atrial Fibrillation	69,941	6.2	81,614	3.5	<0.0001	19,230	24.0	55,519	5.2	<0.0001	32,775	24.4	55,244	2.5	<0.0001
Coronary Artery Disease	116,815	10.3	131,858	5.7	<0.0001	27,217	33.9	96,582	9.0	<0.0001	43,912	32.7	97,497	4.4	<0.0001
Chronic Kidney Disease	110,954	9.8	131,402	5.7	<0.0001	30,083	37.5	88,320	8.3	<0.0001	44,640	33.2	96,039	4.4	<0.0001
Cancer	94,597	8.4	107,868	4.7	<0.0001	17,168	21.4	81,821	7.6	<0.0001	30,587	22.8	84,268	3.8	<0.0001
Diabetes	190,784	16.8	238,642	10.4	<0.0001	30,710	38.3	167,386	15.6	<0.0001	46,934	34.9	201,560	9.2	<0.0001
Hyperlipidemia	342,056	30.2	401,069	17.4	<0.0001	42,251	52.7	310,816	29.0	<0.0001	64,719	48.2	351,457	16.0	<0.0001
Hypertension	436,984	38.6	533,299	23.2	<0.0001	58,478	72.9	392,702	36.7	<0.0001	91,725	68.3	461,706	21.0	<0.0001
Sickle Cell Disease	3,351	0.3	6,337	0.3	0.0007	937	1.2	2,585	0.2	<0.0001	1,983	1.5	4,765	0.2	<0.0001
Sleep Apnea	115,658	10.2	151,215	6.6	<0.0001	145,17	18.1	105,066	9.8	<0.0001	26,125	19.4	132,319	6.0	<0.0001
Anticoagulants†	45,348	4.0	49,202	2.1	<0.0001	11,054	13.8	37,716	3.5	<0.0001	16,511	12.3	37,173	1.7	<0.0001

	All Patients					COVID-19					ARI				
	COVID-19		ARI			Hospitalized		Non-hospitalized			Hospitalized		Non-hospitalized		
Characteristics	N	%	N	%	p-value	N	%	N	%	p-value	N	%	N	%	p-value
Total	**1,132,355**		**2,301,209**			**80,209**		**1,070,125**			**134,392**		**2,198,472**		
**Demographics**															
Antiplatelets‡	33,481	3.0	35,650	1.5	<0.0001	8,481	10.6	27,284	2.5	<0.0001	11,606	8.6	26,736	1.2	<0.0001
Estrogen-Based Birth Control	28,572	2.5	67,163	2.9	<0.0001	489	0.6	28,190	2.6	<0.0001	773	0.6	66,654	3.0	<0.0001
Other Medications that increase risk of stroke§	51,007	4.5	72,375	3.1	<0.0001	12,864	16.0	41,443	3.9	<0.0001	18,531	13.8	58,339	2.7	<0.0001

*The National Patient-Centered Clinical Research Network

†Anticoagulant medications included apixaban, argatroban, betrixaban, dabigatran etexilate, edoxaban, rivaroxaban, and warfarin sodium.

‡Antiplatelet medications included aspirin/dipyridamole, cangrelor, clopidogrel, dipyridamole, prasugrel, ticagrelor, ticlopidine, and vorapaxar sulfate.

§Other Medications that increase risk of stroke included amitriptyline/diazepam /perphenazine, amitriptyline/perphenazine, aripiprazole, asenapine, bevacizumab, bevacizumab-adcd, bevacizumab-bvzr, buserelin, buzepide metiodide/haloperidol, chlorpromazine, chlorpromazined/dipyrone, clozapine, cobicistat/darunavir, cobicistat/darunavir/emtricitabine/tenofovir alafenamide, darunavir, darunavir/cobicistat, decazate, dozine, erythropoietin, fluoxetine/olanzapine, fluphenazine, fluphenazine/nortriptyline, gonadorelin acetate, gonadorelin diacetate tetrahydrate, gonadorelin hydrochloride, gonadorelin/thyrotropin-releasing hormone, goserelin, haloperidol, iloperidone, isopropamide/trifluoperazine, leuprolide, leupronide/norethindrone, loxapine, lurasidone, molindone, nafarelin, nilotinib, nortriptyline/perphenazine, olanzapine, olanzapine/fluoxetine, paliperidone, perphenazine, perphenazine/amitriptyline, phenothiazine, pimavanserin, pimozide, ponatinib, quetiapine, risperidone, tamoxifen, thioridazine, thiothixene, thorazine, tranylcypromine/trifluoperazine, trifluoperazine, triflupromazine, and ziprasidone.

Appendix Table 3. Characteristics of patients diagnosed with COVID-19 or Acute Respiratory Infection (ARI) who experienced a subsequent stroke or thrombotic event* within 31–365 d, in PCORnet[†] April 1, 2022–April 30, 2023.

	All Patients					COVID-19 Patients					ARI Patients				
**Event occurred 31–365 d post index**	COVID-19		ARI			Hospitalized		Non-hospitalized			Hospitalized		Non-hospitalized		
	N	%	N	%	P-value	N	%	N	%	P-value	N	%	N	%	P-value
Number of patients	**17,606**		**21,871**			**3,572**		**14,034**			**5,253**		**16,618**		
Ischemic Stroke	7,221	41	8,765	40.1	N/A	1520	42.6	5,701	40.6	N/A	2,114	40.2	6,651	40.0	N/A
Deep Vein Thrombosis (DVT)	5309	30.2	6781	31.0		1247	34.9	4062	28.9		1878	35.8	4903	29.5	
Hemorrhagic Stroke	2,160	12.3	2,804	12.8		540	15.1	1,620	11.5		924	17.6	1,880	11.3	
Transient Ischemic Attack (TIA)	3,894	22.1	4,659	21.3		512	14.3	3,382	24.1		675	12.8	3,984	24	
Cerebral Venous Sinus Thrombosis	186	1.1	277	1.3		34	1.0	152	1.1		60	1.1	217	1.3	
**Demographics**															
**Age (years)**															
5–17	91	0.5	410	1.9	<0.0001	31	0.9	60	0.4	<0.0001	113	2.2	297	1.8	<0.0001
18–34	501	2.8	935	4.3		128	3.6	373	2.7		225	4.3	710	4.3	
35–49	1,494	8.5	2,250	10.3		256	7.2	1,238	8.8		430	8.2	1,820	11.0	
50–64	4,254	24.2	5,908	27.0		819	22.9	3,435	24.5		1,337	25.5	4,571	27.5	
≥65	11,266	64.0	12,368	56.5		2,338	65.5	8,928	63.6		3,148	59.9	9,220	55.5	
**Sex**															
Female	9,750	55.4	12,833	58.7	<0.0001	1,861	52.1	7,889	56.2	<0.0001	2,695	51.3	10,138	61.0	<0.0001
Male	7,854	44.6	9,035	41.3		1,710	47.9	6,144	43.8		2,557	48.7	6,478	39	
Unknown/ Missing	2	0.0	3	0.0		1	0.0	1	0.0		1	0.0	2	0.0	
**Race**															
American Indian or Alaska Native	62	0.4	160	0.7	<0.0001	15	0.4	47	0.3	<0.0001	31	0.6	129	0.8	<0.0001
Asian and Native Hawaiian or Other Pacific Islander	315	1.8	365	1.7		70	2.0	245	1.8		100	1.9	265	1.6	
Black or African American	2,965	16.8	4,066	18.6		842	23.6	2,123	15.1		1,199	22.8	2,867	17.3	
Missing (No Information, Refuse to Answer, Unknown, Missing)	774	4.4	746	3.4		184	5.2	590	4.2		203	3.9	543	3.3	
Multiple Race or Other	575	3.3	699	3.2		147	4.1	428	3.1		210	4.0	489	2.9	
White	12,915	73.4	15,835	72.4		2,314	64.8	10,601	75.5		3,510	66.8	12,325	74.2	
**Hispanic Ethnicity**															
Yes	1,123	6.4	1,454	6.6	0.4223	295	8.3	828	5.9	<0.0001	392	7.5	1,062	6.4	<0.0001
No	13,680	77.7	17,005	77.8		2,847	79.7	10,833	77.2		4,245	80.8	12,760	76.8	
Other/Missing	2,803	15.9	3,412	15.6		430	12.0	2,373	16.9		616	11.7	2,796	16.8	
**Past Medical History 18 mo before index event**															
Acute COVID-19 Treatments	6,707	38.1	284	1.3	<0.0001	972	27.2	5,735	40.9	<0.0001	87	1.7	197	1.2	0.0086
Alcohol Abuse	658	3.7	929	4.2	0.0103	247	6.9	411	2.9	<0.0001	401	7.6	528	3.2	<0.0001

Event occurred 31–365 d post index	All Patients					COVID-19 Patients					ARI Patients				
	COVID-19		ARI			Hospitalized		Non-hospitalized			Hospitalized		Non-hospitalized		
	N	%	N	%	P-value	N	%	N	%	P-value	N	%	N	%	P-value
Atrial Fibrillation	2,927	16.6	3,754	17.2	0.1555	937	26.2	1,990	14.2	<0.0001	1,497	28.5	2,257	13.6	<0.0001
Coronary Artery Disease	4,885	27.7	6,028	27.6	0.6835	1,485	41.6	3,400	24.2	<0.0001	2,211	42.1	3,817	23	<0.0001
Chronic Kidney Disease	4,880	27.7	5,606	25.6	<0.0001	1,653	46.3	3,227	23.0	<0.0001	2,189	41.7	3,417	20.6	<0.0001
Cancer	3,325	18.9	4,239	19.4	0.1555	898	25.1	2,427	17.3	<0.0001	1,517	28.9	2,722	16.4	<0.0001
Diabetes	5,915	33.6	7,337	33.5	0.9171	1,628	45.6	4,287	30.5	<0.0001	2,268	43.2	5,069	30.5	<0.0001
Hyperlipidemia	9,243	52.5	11,138	50.9	0.0019	2,142	60.0	7,101	50.6	<0.0001	3,159	60.1	7,979	48.0	<0.0001
Hypertension	12,213	69.4	15,060	68.9	0.2757	2,933	82.1	9,280	66.1	<0.0001	4,295	81.8	10,765	64.8	<0.0001
Sickle Cell Disease	111	0.6	173	0.8	0.0606	51	1.4	60	0.4	<0.0001	87	1.7	86	0.5	<0.0001
Sleep Apnea	3,181	18.1	4,119	18.8	0.0515	717	20.1	2,464	17.6	0.0005	1,199	22.8	2,920	17.6	<0.0001
Anticoagulants[‡]	2,098	11.9	2,497	11.4	0.124	599	16.8	1,499	10.7	<0.0001	838	16.0	1,659	10	<0.0001
Antiplatelets[§]	1,594	9.1	1,774	8.1	0.0009	492	13.8	1,102	7.9	<0.0001	545	10.4	1,229	7.4	<0.0001
Estrogen-Based Birth Control	123	0.7	174	0.8	0.2678	12	0.3	111	0.8	0.0036	17	0.3	157	0.9	<0.0001
Other Medications that increase risk of stroke[¶]	1,603	9.1	2,021	9.2	0.6425	567	15.9	1,036	7.4	<0.0001	741	14.1	1,280	7.7	<0.0001

*Cerebral venous sinus thrombosis, deep vein thrombosis, hemorrhagic stroke, ischemic stroke, and transient ischemic attack

[†]The National Patient-Centered Clinical Research Network.

[‡]Anticoagulant medications included apixaban, argatroban, betrixaban, dabigatran etexilate, edoxaban, rivaroxaban, and warfarin sodium.

[§]Antiplatelet medications included aspirin/dipyridamole, cangrelor, clopidogrel, dipyridamole, prasugrel, ticagrelor, ticlopidine, and vorapaxar sulfate.

[¶]Other Medications that increase risk of stroke included amitriptyline/diazepam /perphenazine, amitriptyline/perphenazine, aripiprazole, asenapine, bevacizumab, bevacizumab-adcd, bevacizumab-bvzr, buserelin, buzepide metiodide/haloperidol, chlorpromazine, chlorpromazined/dipyrone, clozapine, cobicistat/darunavir, cobicistat/darunavir/emtricitabine/tenofovir alafenamide, darunavir, darunavir/cobicistat, decazate, dozine, erythropoietin, fluoxetine/olanzapine, fluphenazine, fluphenazine/nortriptyline, gonadorelin acetate, gonadorelin diacetate tetrahydrate, gonadorelin hydrochloride, gonadorelin/thyrotropin-releasing hormone, goserelin, haloperidol, iloperidone, isopropamide/trifluoperazine, leuprolide, leupronide/norethindrone, loxapine, lurasidone, molindone, nafarelin, nilotinib, nortriptyline/perphenazine, olanzapine, olanzapine/fluoxetine, paliperidone, perphenazine, perphenazine/amitriptyline, phenothiazine, pimavanserin, pimozide, ponatinib, quetiapine, risperidone, tamoxifen, thioridazine, thiothixene, thorazine, tranylcypromine/trifluoperazine, trifluoperazine, triflupromazine, and ziprasidone.

Appendix Table 4. Incidence per 10,000 patients with Stroke and Thrombotic Event Subtypes among patients with COVID-19 or Acute Respiratory Illness (ARI) by hospitalization status and days from acute illness adjusted by 30-d time periods*

	All Patients		Hospitalized Patients		Non-hospitalized Patients	
	COVID-19	ARI	COVID-19	ARI	COVID-19	ARI
Events and timelines	20	13	81	73	16	10
All events **31–90 d** and no record in 18 mo prior						
Ischemic Stroke	8	5	30	27	6	4
Deep Vein Thrombosis (DVT)	7	4	31	28	5	3
Hemorrhagic Stroke	2	2	12	12	2	1
TIA	4	2	9	8	3	2
Cerebral Venous Sinus Thrombosis	0	0	1	1	0	0
All events **91–180 d** and no record in 18 mo prior	19	12	57	51	15	9
Ischemic Stroke	8	5	24	19	6	4
Deep Vein Thrombosis (DVT)	6	4	20	19	5	3
Hemorrhagic Stroke	2	1	7	8	2	1
TIA	4	2	7	6	3	2
Cerebral Venous Sinus Thrombosis	0	0	1	1	0	0
All events **181–365 d** and no record in 18 mo prior	16	10	38	34	14	8
Ischemic Stroke	7	4	17	14	6	3
Deep Vein Thrombosis (DVT)	5	3	12	11	4	2
Hemorrhagic Stroke	2	1	5	6	2	1
TIA	4	2	6	4	3	2
Cerebral Venous Sinus Thrombosis	0	0	0	0	0	0
All events **31–365 d** and no record in 18 mo prior	14	9	40	35	12	7
Ischemic Stroke	6	3	17	14	5	3
Deep Vein Thrombosis (DVT)	4	3	14	13	3	2
Hemorrhagic Stroke	2	1	6	6	1	1
TIA	3	2	6	4	3	2
Cerebral Venous Sinus Thrombosis	0	0	0	0	0	0

*Incidence per 30 d calculated by dividing 31–90, 91–180, 181–365, and 31–365 daytime periods by the number of 30-d time periods each included.
